# Exploring multidimensional characteristics in cervicogenic headache: Relations between pain processing, lifestyle, and psychosocial factors

**DOI:** 10.1002/brb3.2339

**Published:** 2021-09-02

**Authors:** Sarah Mingels, Wim Dankaerts, Ludo van Etten, Liesbeth Bruckers, Marita Granitzer

**Affiliations:** ^1^ REVAL Rehabilitation Research Centre, Faculty of Rehabilitation Sciences and Physiotherapy Hasselt University Hasselt Belgium; ^2^ Musculoskeletal Research Unit, Department of Rehabilitation Sciences Faculty of Kinesiology and Rehabilitation Sciences Leuven University Leuven Belgium; ^3^ Department of Biometrics Zuyd Hogeschool Heerlen The Netherlands; ^4^ Interuniversity Institute for Biostatistics and Statistical Bioinformatics Hasselt University Hasselt Belgium

**Keywords:** cervicogenic headache, lifestyle, pain, psychology

## Abstract

**Objective:**

Although multidimensional interventions including physiotherapy, psychology, and education are generally recommended in managing headache, and to prevent chronification, such approach is lacking in cervicogenic headache (CeH). Therefore, exploring CeH within a biopsychosocial framework is deemed an essential first step.

**Methods:**

Non‐randomized cross‐sectional design to compare pain processing, lifestyle, and psychosocial characteristics between 18 participants with CeH (CeH group) (40.2 ± 10.9 years) and 18 matched controls (control group) (39.2 ± 13.1 years). Pain processing characteristics included degree of central sensitization (Central Sensitization Inventory), and (extra)‐cephalic pressure pain thresholds (kPa/cm²/s). Lifestyle characteristics included sleep quality (Pittsburgh Sleep Quality Index), physical activity, screen time, and sedentary time (hours a week). Psychosocial characteristics included degree of depression, anxiety and stress (Depression Anxiety Stress Scale‐21), and quality of life (Headache Impact Test‐6).

**Results:**

*Pain processing characteristics*: More (*p* = .04) participants in the CeH group showed higher degrees of central sensitization compared to the control group. Lower (*p* < .05) (extra)‐cephalic pressure pain thresholds were revealed in the CeH group compared to the control group for each muscle. *Lifestyle and psychosocial characteristics*: Compared to the control group, sleep quality and headache‐related quality of life were worse (*p* < .0001) in the CeH group. Severe to extreme stress was experienced by more participants in the CeH group (*p* = .02). Further, significant relations between pain processing and (1) lifestyle characteristics and (2) psychosocial characteristics were seen in the CeH group.

**Conclusion:**

Exploring multidimensional characteristics in CeH exposed relations between pain processing, lifestyle, and psychosocial characteristics. These novel findings fill a gap in the current scientific literature, and highlight the need for outcome research targeting lifestyle and psychosocial factors.

## Abstract 2

Cervicogenic headache is a complex headache disorder. Pain processing, lifestyle, and psychosocial characteristics interact in patients with cervicogenic headache.

## 1 INTRODUCTION

The International Classification of Headache Disorders 3 (ICHD) defines cervicogenic headache (CeH) as: “Headache caused by a disorder of the cervical spine and its components (bony, disc and/or soft tissue elements) usually but not invariably accompanied by neck pain” (Headache Classification Committee of the International Headache Society, 2013). General recommendations for the non‐pharmacological non‐invasive management of CeH can be summarized as: Manual therapy applied to the cervical and thoracic spine whether or not combined with low load endurance exercises, trigger point therapy, combination of static and dynamic cervical, scapulothoracic strengthening and endurance exercises, and low‐load craniocervical and cervicoscapular endurance exercises (Côté et al., 2019; Fernandez et al., 2020; Gross et al., 2016; Luedtke et al., 2016; Varatharajan et al., 2016). Despite these recommendations, there is limited evidence regarding the effectiveness of such interventions on pain intensity, frequency, duration, and disability in the long‐term (Fernandez et al., 2020). Further, results from a systematic review with meta‐analysis revealed that pooling results of studies on manually managing CeH did not support significant effects of such therapies on headache‐intensity, ‐disability, and ‐frequency (Coelho et al., 2019). Based on these inconsistencies it was already proposed that not all patients with CeH benefit from particular interventions, and that not all interventions are appropriate (Fernández‐De‐Las‐Peñas & Cuadrado, 2014).

Although non‐pharmacological interventions could play an important role in managing CeH, scientific evidence for success of such interventions remains contradictory (Coelho et al., 2019; Falsiroli Maistrello et al., 2019). One in four patients with CeH is non‐responsive to musculoskeletal physiotherapy. According to physiotherapists, such non‐responsiveness can be related to augmented pain processing in the central nervous system, and presence of negative psychosocial factors (Liebert et al., 2013; Moore et al., 2017). It was recently stated that local therapy, exclusively addressing the upper‐cervical spine, was ineffective if signs of central sensitization (CS) are already present. In such state, local therapy might act as a minimal peripheral nociceptive stimulus maintaining CS (Fernández‐De‐Las‐Peñas et al., 2020; Woolf, 2011). Headaches which are merely mediated by a peripheral nociceptive source, also known as bottom‐up sensitizer (e.g., musculoskeletal disorder of the upper‐cervical), can be addressed by targeting that peripheral source through manual therapy and/or local exercises (Fernández‐De‐Las‐Peñas et al., 2020). However, if additionally central mechanisms, also known as top‐down sensitizers (e.g., anxiety, stress), are involved, clinical management needs to shift towards a multimodal approach including physical, psychological, cognitive, lifestyle and educational dimensions directed at normalizing CS (Bialosky et al., 2018; Fernández‐De‐Las‐Peñas et al., 2020). Such dimensions are known influencers on therapy‐responsiveness in other musculoskeletal syndromes such as non‐specific chronic low back pain (O'sullivan, 2012). Integrating both bottom‐up and top‐down interventions in order to modulate pain at different levels of the nervous system therefore seems advised in complex pain disorders such as CeH (Fernández‐De‐Las‐Peñas et al., 2020; Lluch Girbés et al., 2015). Disturbed pain processing has already been reported in patients with chronic CeH (Chua et al., 2011). However, it is yet to determine if such disturbances are also present in patients with episodic CeH, and the factors which influence it. Identifying maladaptive pain processing in episodic CeH is relevant in the context of headache chronification since headache‐frequency is related to sensitization (Buchgreitz et al., 2006).

Yet, scientific results on relations between pain processing, lifestyle and psychosocial characteristics are, to the best of our knowledge, lacking in episodic CeH. Such relations however need to be explored since they relate to chronification of pain (Borsook et al., 2018). To fill this gap, we propose to evaluate patients with episodic CeH within a biopsychosocial framework. Therefore, the aim of this study was to analyze relations between pain processing, lifestyle, and psychosocial characteristics in patients with episodic CeH.

## 2 METHODS

### 2.1 Design

This study is designed as follows:

Non‐randomized cross‐sectional comparison of pain processing, lifestyle, and psychosocial characteristics between a CeH group and matched healthy control group;

Analysis of relations between pain processing, lifestyle, and psychosocial characteristics within a CeH group and matched healthy control group.

### 2.2 Sample size

Sample size calculation was not performed to establish a treatment‐effect, but rather to assess feasibility in the context of an exploratory study. A priori sample size calculation (G*Power 3.1.9.4, Kiel Germany) to detect differences between groups, based on cervical pressure pain thresholds (PPT) (mean, standard deviation), resulted in a required sample size of 15 participants per group (power 80%; *α* = .05) (Chua et al., 2011).

### 2.3 Participants and ethics

The neurological staff of the headache department of the AZ Vesalius hospital (Belgium) identified and referred participants meeting the study's inclusion criteria for episodic CeH between June 2018 (start enrolment) and July 2019 (end enrolment). Additionally, a general call was launched at Hasselt University and Zuyd Hogeschool (January 2018 to July 2019). Potential participants for the control group were recruited (January 2018 to August 2019) by convenience sampling, word‐of‐mouth advertising within Zuyd Hogeschool, and the personal network of the involved researcher.

*Inclusion criteria for the CeH group* were: Dutch‐speaking participants between 18 and 55 years, with body mass index (BMI) between 18.5 and 24.9 kg/m² (Tashani et al., 2017), diagnosed with secondary episodic CeH according to the ICHD‐3 (Table 1) by a neurologist (Headache Classification Committee of the International Headache Society, 2013), having a score of 30 on the 11‐item Mini Mental State Examination test (scale of 0 to 30) indicating normal cognitive capacity. *Inclusion criteria for the control group* were: Dutch‐speaking asymptomatic healthy participants between 18 and 55 years, with BMI between 18.5 and 24.9 kg/m² (Tashani et al., 2017), having a score of 30 on the 11‐item Mini Mental State Examination test (scale of 0 to 30) indicating normal cognitive capacity.

**TABLE 1:** International Classification for Headache Disorders 3 diagnostic criteria for CeH

Diagnostic criteria	
A	Any headache fulfilling criterion C
B	Clinical and/or imaging evidence of a disorder or lesion within the cervical spine or soft tissues of the neck, known to be able to cause headache
C	Evidence of causation demonstrated by at least two of the following: 1. Headache has developed in temporal relation to the onset of the cervical disorder or appearance of the lesion 2. Headache has significantly improved or resolved in parallel with improvement in or resolution of the cervical disorder or lesion 3. Cervical range of motion is reduced and headache is made significantly worse by provocative maneuvers 4. Headache is abolished following diagnostic blockade of a cervical structure or its nerve supply
D	Not better accounted for by another ICHD‐3 diagnosis

**TABLE 2:** Demographics and group characteristics of the CeH group (*n* = 18) and control group (*n* = 18)

	CeH group	Control group	*p*
Age (y), mean (SD) [CI]	40.2 (10.9) [34.6;45.8]	39.2 (13.1) [32.7;45.7]	.80^a^
BMI (kg/m²), mean (SD) [CI]	23.5 (3.2) [21.9;25.1]	23.2 (3.2) [21.6;24.8]	.76^a^
Marital status, *n* (%) Married Living together In a relation (not living together) Single	9 (50) 5 (27.8) 2 (11.1) 2 (11.1)	9 (50) 4 (22.2) 3 (16.7) 2 (11.1)	1^b^
Socioeconomic status, *n* (%) *Employment* Student Working Services Self‐employed *Level of education* Secondary studies Graduate school or university	2 (11.1) 16 (88.9) 14 (87.5) 2 (12.5) 2 (11.1) 16 (88.9)	3 (16.7) 15 (83.3) 13 (72.2) 2 (12.5) 2 (11.1) 16 (88.9)	.65^b^ 1^b^
Dominant hand, *n* (%) Left Right	3 (16.7) 15 (83.3)	0 18 (100)	.22^b^

Abbreviations: *n*, number participants; y, years.

^a^Unpaired *t*‐test; ^b^Contingency table for categorical variables (Fisher's exact test).

**TABLE 3:** Headache characteristics of the CeH group (*n* = 18)

Headache characteristics	Result
Headache duration, mean hours/episode (SD) [CI]	4.1 (1.6) [3.3;4.9]
General headache intensity, mean VAS/episode (SD) [CI]	61 (14) [54.4;67.4]
Headache‐frequency, median days/month [IQR]	11 [10;15.8]
Referred pain from the neck, *n* (%) Yes No	18 (100) 0
Instantaneous headache‐intensity Mean NPRS (SD) [CI] Instantaneous neck‐pain‐intensity Mean NPRS (SD) [CI]	0.7 (1) [0.3;1.2] 0

*n* = number participants; VAS = 100 mm Visual Analogue Scale; NPRS = 11‐point Numeric Pain Rating Scale; IQR = 25–75% interquartile range.

**TABLE 4:** Summary: CSI and PPTs in the CeH group (*n* = 18) and control group (*n* = 18)

Central sensitization inventory			
Dimensions, *n* (%)	CeH group	Control group	*p* ^a^ (OR)
Subclinical (0–29) Mild (30–39) Moderate (40–49) Hard (50–59) Extreme (60–100)	4 (22.2) 5 (27.8) 7 (38.9) 2 (11.1) 0	10 (55.6) 3 (16.7) 5 (27.8) 0 0	.18
Subclinical vs. mild, moderate, hard extreme combined	4 vs. 14	10 vs. 8	**.04 (4.4)**

Pressure pain threshold			
Muscle, kPa/cm² (SD) [CI]	**CeH group**	**Control group**	***p* ^b^ (ES)**
Suboccipital left	206.5 (78.8) [166.2;246.8]	332.6 (268.2) [199.3;466]	**.03** (0.64)
Suboccipital right	179.3 (75.1) [140.8;217.7]	273.3 (203.2) [172.3;374.4]	**.009** (0.61)
Erector spine left	428.1 (236.2) [310.6;545.5]	584 (229.6) [469.9;698.2]	**.04** (0.67)
Erector spine right	418 (185) [323.3;512.7]	596.2 (214.3) [489.6;702.8]	**.008** (0.89)
Tibialis anterior left	365.3 (122.9) [302.3;425.2]	613.2 (263.1) [482.4;744]	**.001** (1.21)
Tibialis anterior right	397.3 (144.2) [323.5;471.1]	626.9 (284.4) [485.5;768.4]	**.007** (1.03)

Bold numbers = *p* < .05.

Abbreviations: ES, effect size; *n*, number participants; OR, odds ratio.

^a^Contingency table for categorical variables (Fisher's exact test); ^b^Unpaired *t*‐test.

*Exclusion criteria* for both groups were: Pregnancy, physiotherapy for head‐ or neck‐related disorders in the past month before the start of the study, serious pathology (musculoskeletal, neurological, endocrine, cardiovascular, psychiatric), pain radiation to the arm(s), medication overuse (intake of NSAID's, opioids, acetylsalicylic acid, triptans, simple analgesics for >10 days/month >3 months), history of neck/head trauma, orthodontics. Eligible participants had to be able to understand, read, and sign the informed consent before officially being enrolled.

Nineteen participants were recruited and selected to compose the CeH group. These participants were given a four‐week headache‐diary. The control group was matched for age, gender, ethnicity, and socio‐economic status (level of education, employment). Two participants (one from the CeH group, one from the control group) had to be excluded after the measurements because of technical artefacts, resulting in 18 eligible participants per group.

The study, which was part of a larger project (hence the larger sample size), was registered as an observational study at ClinicalTrials.gov (NCT02887638). The ‘Medisch Ethische ToetsingsCommissie’ of Zuyderland and Zuyd Hogeschool (NL. 55720.09615), and the ‘Comité Medische Ethiek’ of the Ziekenhuis Oost‐Limburg (B371201423025) granted their approval. Protection of personal data is legally determined by the Belgian law of December 8, 1992. All test procedures involving human participants were in accordance with the ethical standards of the institutional research committees and the 1964 Helsinki Declaration and its later amendments.

### 2.4 Outcomes, measurements, and instruments

Headache, pain processing, lifestyle, and psychosocial characteristics were the outcomes of interest.

#### 2.4.1 Headache characteristics

Headache characteristics, that is, headache‐intensity (mean 100 mm Visual Analogue Scale (VAS) per attack during a month), duration (mean hours per attack during a month), and frequency (days per month) were extracted from the headache diary of the Belgian Headache Society (2020). Additional information on referred pain from the neck (yes/no) was obtained through a customized questionnaire and anamnesis.

##### 2.4.1.1 Pain processing characteristics

*Symptoms of central sensitization* were identified using the Dutch Central Sensitization Inventory (CSI) (Kregel et al., 2016). Test–retest reliability (ICC 0.82–0.97), and internal constancy (Cronbach's *α* 0.87–0.91) are good to excellent (Scerbo et al., 2018). The CSI is a self‐reported screening tool in which participants determine to what extent statements apply to him/her. Part A was used for further analysis: 25 health‐related somatic and emotional symptoms scored on a 0–4 Likert scale (“never,” “rarely,” “sometimes,” “often,” “always”) with a maximum score of 100. CSI‐scores 0–29 indicate subclinical, 30–39 mild, 40–49 moderate, 50–59 hard, and 60–100 extreme symptoms of central sensitization (Neblett et al., 2017).

PPTs (kPa/cm²/s) of the bilateral suboccipitals, erector spine at L1, and tibialis anterior were measured with an electronic pressure algometer (Somedic AB, Stockholm, Sweden) (Balaguier et al., 2016; Castien et al., 2018; Koppenhaver et al., 2015; Walton et al., 2011; Ylinen et al., 2007). PPT is defined as the minimal amount of pressure that elicits pain. Hypersensitivity over remote, extra‐cephalic sites is considered as a sign of central sensitization. Intra‐rater reliability of cervical PPT‐measurements are moderate to good (ICC 0.79–0.90) in healthy participants, and good to excellent (ICC 0.82–0.99) in participants with headache (Balaguier et al., 2016; Martínez‐Segura et al., 2012; Walton et al., 2011). Intra‐rater reliability of erector spine PPT‐measurements are excellent (ICC 0.91 ± 0.07) in healthy participants (Binderup et al., 2010). Intra‐rater reliability of tibialis anterior PPT‐measurements are excellent in healthy participants (ICC 0.94), and patients with neck pain (ICC 0.97) (Walton et al., 2011).

#### 2.4.2 Lifestyle characteristics

S*leep quality* was assessed via the Dutch Pittsburgh Sleep Quality Index (PSQI) which is a standardized, valid, and reliable self‐reported one‐month recall questionnaire (Marinus et al., 2003; Mollayeva et al., 2016). We refer to the systematic review and meta‐analysis by Mollayeva et al. (2016) concerning the psychometric properties. The index differentiates poor from good sleepers by measuring seven components: Subjective sleep quality, sleep latency, sleep duration, habitual sleep efficiency, sleep disturbances, use of sleeping medication, and daytime dysfunction. Scores on each of these components vary from 0 (no problem) to 3 (serious problem). A maximum score exceeding 5/21 indicates poor sleep quality (Buysse et al., 1989; Smyth, 2008).

*Physical activity* (mean hours a week), *screen‐time* (mean hours a week computer‐use), and *sedentary‐time* during free‐time and work (mean hours a week) were extracted from a customized one‐week recall questionnaire.

#### 2.4.3 Psychosocial characteristics

The degree of *depression, anxiety and/or stress* was estimated by the Dutch Depression Anxiety Stress Scale‐21 (DASS‐21), a valid self‐reported one‐week recall questionnaire (de Beurs et al., 2001; Parkitny et al., 2012). Internal constancy for the three subscales is good to excellent (Cronbach's *α* 0.91, 0.84, and 0.90, respectively) (de Beurs et al., 2001; Parkitny et al., 2012). Each of the three sub‐scales contains seven items. The depression subscale assesses dysphoria, hopelessness, devaluation of life, self‐deprecation, lack of interest, anhedonia, and inertia. The anxiety subscale estimates autonomic arousal, skeletal muscle effects, situational anxiety, and subjective experience of anxious affect. The stress subscale evaluates difficulty in relaxing, nervous arousal, and being easily upset and impatient. Items are scored on a Likert‐scale (0 = “Did not apply to me at all,” and 3 = “Applied to me very much or most of the time”). Scores for depression, anxiety, and stress were calculated by summing the scores for the relevant items: subscale depression: 3,5,10,13,16,17,21; subscale anxiety: 2,4,7,9,15,19,20, and subscale stress: 1,6,8,11,12,14,18. We refer to Lovibond and Lovibond (1995) for interpretation of the scores.

Impact *of headache on quality of life* was assessed with the Dutch Headache Impact Test‐6 (HIT‐6) (Buse et al., 2012). The HIT‐6 shows good test re‐test reliability (ICC 0.80), and internal consistency (Cronbach's *α* 0.79) (Kosinski et al., 2003; Martin et al., 2004). The HIT‐6 evaluates the impact of headache on daily activities: the ability to function at work, school, home, and in social situations. Questions are answered by: “never,” “rarely,” “sometimes,” “very often,” and “always,” scores are 6, 8, 10, 11, and 13, respectively. The impact of headache on daily life depends on the total score, which varies between 36 and 78. Scores are interpreted as follows: ≤49 means no to little impact of headache on daily life, between 50 and 55 indicates that headache seems to affect daily life, between 56 and 59 means headache has a significant impact on daily life, and ≥60 indicates that headache has a very heavy impact on daily life (Kawata et al., 2005; Martin et al., 2004).

### 2.5 Test procedure

A condition to be measured was a score of <3 on the 11‐point Numeric Pain Rating Scale on the test day. Participants were asked not to take analgesics, muscle relaxants, and caffeine‐containing beverages 24 h prior to the measurements. Prophylactic treatment(s) remained unchanged (Headache Classification Committee of the International Headache Society, 2013). Measurements were performed in a real‐life set‐up with a constant room temperature of 25°C at the Motion Lab (Zuyd Hogeschool, The Netherlands), and executed by the main researcher.

Questionnaires were completed before PPT measurements. The questionnaires used allow all kinds of health practitioners (e.g., physiotherapists) to screen for psychosocial risk factors in patients with musculoskeletal pain (Matheson et al., 2020).

A standardized protocol was used to measure PPTs of the bilateral suboccipitals, erector spine at L1 (neutral prone position) and tibialis anterior (seated with 80° knee‐flexion) (Koppenhaver et al., 2015; Walton et al., 2011). Pressure was perpendicularly applied directly on the muscle belly, staring at 0 to maximal 1000 kPa, using a 1 cm² probe with a slope of 30 kPa/s (Alburquerque‐Sendín et al., 2018; O'sullivan et al., 2014). Participants were instructed to push the stop button when the sensation of pressure first changed into pain. An exercise trial was performed once on the right thigh before actually measuring. Measurements were executed twice (ICC 0.86–0.99) after a five‐minute interval in a standardized column‐wise order: Suboccipital left, erector spine at L1 left, tibialis anterior left, suboccipital right, erector spine at L1 right, and tibialis anterior right (Balaguier et al., 2016; Finocchietti et al., 2011; O'sullivan et al., 2014; Prushansky et al., 2004). Mean values of two measurements on each location were used for further statistical analysis (Chesterton et al., 2007; Walton et al., 2011). The principal researcher performed the test procedure for both the CeH group and control group. All outcomes were evaluated by the principal researcher (physiotherapist with degree in manual therapy, >10‐year experience).

### 2.6 Research questions and statistical analyses

Analysis was completed via SAS JMP Pro 14. Two‐tailed tests at 5% level of significance were reported. *Demographics and group characteristics*: Continuous outcomes were compared between groups by applying unpaired *t*‐tests (conditions of normality and equality of variances were met). Contingency tables (Fisher's exact test) were composed to analyze distributions of categorical variables (proportions) between groups.

*Relations* between the independent variable's age, BMI, their interaction (age*BMI) (continuous), socioeconomic status (categorical), and dependent pain processing, lifestyle, and psychosocial outcome variables were evaluated via stepwise multiple linear or ordinal regression to obtain the best model fit (i.e., smallest mean square of the error, Variance Inflation Factor (VIF) < 4). Conditions to apply such models (normality, homoscedasticity, no collinearity, linearity in multiple linear regression, and test of parallel lines *p* > .05 in ordinal regression) had to be met. Cut‐off for multicollinearity was set at VIF ≥ 4. Tukey corrections for multiple testing were applied.

*Headache characteristics*: Intensity, duration, frequency, and neck pain were descriptively presented as means (SD), interquartile range, and proportions (%).

*Research question 1*: Do participants with episodic CeH show signs and symptoms of disturbed pain processing compared to the control group?

The categorical version of CSI (five classes) was proportionally presented (%). Contingency tables (Fisher's exact test) were composed to compare distributions of results between groups. Groups were compared in terms of the five classes CSI, and also for the two‐class‐version (Fisher's exact test) with subclinical versus the other classes (i.e., mild, moderate, hard, and extreme combined).

PPTs were presented as means (SD). Unpaired *t*‐tests (conditions of normality and equality of variances were met) were used to compare average cephalic and extra‐cephalic PPTs between groups. Effect sizes (ES) to quantify the magnitude in mean PPT‐difference between groups (Cohen's *d*) were reported and interpreted as: ≤0.2 small, 0.21 to 0.49 moderate, 0.50 to 0.79 medium, and ≥0.8 large ES (Rosenthal, 1996).

*Research Question 2*: Do participants with episodic CeH present with different lifestyle and/or psychosocial characteristics compared to the control group?

Distributions of results on the PSQI (four classes), level of physical activity (four classes), screen‐time (five classes), sedentary‐time (three classes), DASS‐21 (four classes), and HIT‐6 (four classes) were proportionally presented (%), and compared between groups by composing contingency tables (Fisher's exact test). Sleep quality (10 cm VAS) and sleep duration (hours/night) were presented as means (SD), and compared between groups by using the Mann–Whitney test.

*Research Question 3*: Are headache, pain processing, lifestyle, and psychosocial characteristics related in both groups?

Linear regression models (simple, multiple, ordinal) or contingency tables (Fisher's exact test) were composed depending on the dependent and independent variables, and the conditions (cfr. described above) to analyze possible relations. Variables were selected based on a priori hypotheses and entered in the regression model, leading to many hypotheses being tested. A backward stepwise approach was used to downsize the model and obtain the best model fit (i.e., smallest mean square of the error, VIF < 4).

## 3 RESULTS

### 3.1 Demographics and group characteristics (Table 2)

Demographics and group characteristics were comparable between groups. Age, BMI, their interaction (age*BMI), level of education, and employment did not significantly influence pain processing, lifestyle, or psychosocial outcome variables (appendix a).

### 3.2 Headache characteristics (Table 3)

Participants with CeH suffered from an episodic, moderate to severe intense headache with a mean duration of 4.1 hours/episode. Referred pain from the neck was reported by all 18 participants. Headache or neck pain was not present at the start of the test procedure.

### 3.3 Pain processing characteristics (Table 4)

*CSI*: No significant differences were seen between groups for the ordinal version of the CSI. However, transforming the ordinal to a binary CSI scale (i.e., subclinical vs. mild, moderate, hard extreme combined) revealed that significantly more (*p* = .04, Odds Ratio 4.4) participants in the CeH group suffered from a higher degree of symptoms of CS compared to participants in the control group.

*PPTs*: Comparing absolute PPTs between groups revealed significantly lower cephalic and extra‐cephalic PPTs in the CeH group compared to the control group for each muscle (*p* < .05). ES, ranging between 0.61 and 1.21, were medium to large.

### 3.4 Lifestyle and psychosocial characteristics (Table 5)

*Lifestyle characteristics*: The recommended level of weekly physical activity (i.e., “high” in Table 5) was achieved by 11.1% of participants in the CeH group, and by 16.7% in the control group (Global Strategy on Diet, Physical Activity & Health, 2016). Sleep quality, measured with the 10 cm VAS, was significantly worse (*p* < .0001) in the CeH group. Screen‐time, sedentary‐time, and distribution of proportions on the PSQI did not differ significantly between groups.

**TABLE 5:** Lifestyle and psychosocial characteristics in the CeH group (*n* = 18) and control group (*n* = 18)

Lifestyle characteristics	CeH group	Control group	*p* ^a^ (ES)
Pittsburgh Sleep Quality Index, *n* (%) Optimal (≤5) Borderline (6–7) Poor (≥8)	7 (38.9) 5 (27.8) 6 (33.3)	11 (61.1) 3 (16.7) 4 (22.2)	.5
Sleep quality (10 cm VAS), mean (SD) [CI]	6.6 (1.2) [5.9;7.2]	2.6 (2.2) [1.5;3.7]	<**.0001** ^b^ (0.95)
Sleep duration (hours/night), mean (SD) [CI]	6.9 (0.9) [6.5;7.4]	7.4 (1.4) [6.7;8.1]	.25^b^
Physical activity hours/week, *n* (%) Low (≤2 h) Moderate (1 to 2 times, ≥30 min) High (minimal 3 times, ≥30 min)	7 (38.8) 9 (50) 2 (11.1)	13 (72.2) 2 (11.1) 3 (16.7)	.05
Screen‐time hours/week, *n* (%) Little (<7 h) Substantiate (7–14 h) Moderate (14–21 h) Severe (>21 h)	1 (5.6) 5 (27.8) 1 (5.6) 11 (61.1)	1 (5.6) 6 (33.3) 2 (11.1) 9 (50)	.89
Sedentary‐time: free‐time hours/day, *n* (%) No (0 h) Little (<3 h) Moderate (3–6 h) Severe (≥7 h)	1 (5.6) 7 (38.9) 9 (50) 1 (5.6)	0 4 (22.2) 13 (72.2) 1 (5.6)	.63
Sedentary‐time: work hours/day, *n* (%) No (0 h) Little (<3 h) Moderate (3–6 h) Severe (≥7 h)	1 (5.6) 3 (16.7) 7 (38.9) 7 (38.9)	1 (5.6) 1 (5.6) 10 (55.6) 6 (33.3)	.62
**Psychosocial characteristics**	**CeH group**	**Control group**	***p* ^a^**
Depression, Anxiety, Stress Scale‐21, *n* (%)			
Depression	10 (55.6)	14 (77.8)	.33
Normal (0–9)	2 (11.1)	0	.82
Mild (10–13)	2 (11.1)	3 (16.7)	**.01**
Moderate (14–20)	3 (16.7)	1 (5.6)	
Severe (21–27)	1 (5.6)	0	
Extreme (≥28)	7 (38.9)	9 (50)	
Anxiety	6 (33.3)	5 (27.8)	
Normal (0–7)	2 (11.1)	3 (16.7)	
Mild (8–9)	1 (5.6)	1 (5.6)	
Moderate (10–14)	2 (11.1)	0	
Severe (15–19)	7 (38.9)	7 (38.9)	
Extreme (≥20)	4 (22.2)	3 (16.7)	
Stress	1 (5.6)	8 (44.4)	
Normal (0–14)	3 (16.7)	0	
Mild (15–18)	3 (16.7)	0	
Extreme (≥34)			
Headache Impact Test‐6, *n* (%)			
Little/none (≤49)	2 (11.1)	18 (100)	<**.0001**
Substantiate (50–55)	1 (5.6)	0	
Moderate (56–59)	6 (33.3)	0	
Severe (>60)	9 (50)	0	
*p* ^a^ within the group	**.008**	<**.0001**	

Bold numbers = *p* < .05.

Abbreviations: ES, effect size; *n*, number participants; VAS, 10 cm Visual Analogue Scale (0 = best, 10 = worst sleep quality).

^a^Contingency table for categorical variables (Fisher's exact test); ^b^Mann–Whitney test.

*Psychosocial characteristics*: Significantly more (*p* = .008) participants in the CeH group reported a severe impact of headache on their quality of life (50%), compared to only 11.1% of participants reporting little to no impact. Distribution of scores on the ordinal HIT‐6 differed significantly (*p* < .0001) between groups. Headache‐related quality of life was significantly worse in the CeH group, with 83.3% of participants reporting a moderate to severe impact of headache on their quality of life compared to 0% of participants in the control group. Distribution of scores on the ordinal DASS‐stress differed significantly (*p* = .02) between groups. Severe to extreme stress was experienced by 33.4% of participants in the CeH group compared to 0% of participants in control group.

No significant differences between the groups were seen concerning the DASS‐depression and DASS‐anxiety.

### 3.5 Relations between headache, pain processing, lifestyle, and psychosocial characteristics (Table 6)

Following Table 6 summarizes significant relations between: Lifestyle and headache characteristics, psychosocial and headache characteristics, pain processing and headache characteristics in the CeH group, and between pain processing and lifestyle characteristics, psychosocial, and pain processing characteristics in both groups.

**TABLE 6:** Summary of significant relations between headache, pain processing, lifestyle, and psychosocial characteristics in the CeH group (*n* = 18) and control group (*n* = 18)

Relation	CeH group	Control group
Lifestyle (independent) and headache characteristics (outcome) Sitting‐time at work related to headache‐frequency Screen‐time related to headache‐intensity	Estimate 1.17, *p* = .04* Estimate 0.78, *p* = .04*	N/A
Psychosocial (independent) and headache characteristics (outcome) DASS‐anxiety related to headache‐frequency^a^	Estimate 17.5, *p* = .04*	N/A
Pain processing (independent) and headache characteristics (outcome) Right suboccipital PPTs related to headache‐intensity Left erector spine PPTs related to headache‐intensity Right tibialis anterior PPTs related to headache‐duration	Estimate −0.01, *p* = .04* Estimate −0.004, *p* = .04* Estimate −0.007, *p* = .02*	N/A
Pain Processing (independent) and lifestyle characteristics (outcome) CSI related to HIT‐6^b^	*p* < .0001**	N/A
Lifestyle (independent) and pain processing characteristics (outcome) Sleep quality related to CSI^c^ Level of physical activity related to right suboccipital PPTs^d^ Screen‐time related to right suboccipital PPTs^e^ Sedentary‐time at work related to left erector spine PPTs Screen‐time related to right tibilialis anterior PPTs^Δ^ Level of physical activity related to left tibialis anterior PPTs^ΔΔ^	*p* = .002*** Estimate 159.36, *p* = .005* Estimate 84.74, *p* = .04* Estimate −0.001, *p* = .69* Estimate −148.71, *p* = .20* Estimate 2.31, *p* = .94*	*p* = .11*** Estimate −308.43, *p* = .28* Estimate 101,55, *p* = .41* Estimate −38.73, *p* = .04* Estimate 124.8, *p* = .04* Estimate −188.4, *p* = .04*
Psychosocial (independent) and pain processing characteristics (outcome) DASS‐stress + PSQI related to left suboccipital PPTs DASS‐stress + HIT‐6 related to right suboccipital PPTs DASS‐stress related to right tibialis anterior PPTs DASS‐stress related to CSI^f^ PSQI related to CSI^g^ HIT‐6 related to left tibialis anterior PPTs	Estimates: DASS −94.07, PSQI −11.61, *p* = .004* (VIF 1.82) Estimates: DASS −60.28, HIT‐6 −4.86, *p* = .007* (VIF 1.01) Estimate: −95.22, *p* = .004* *p* = .005*** *p* = .46*** Estimates HIT‐6 0: 146.5, 1: 34, 2: 25.3, 3: −54.67), *p* = .02*	Estimates: DASS 6.02, PSQI 14.75 (VIF 1), *p* = .61* N/A Estimate: 14.99, *p* = .1* *p* = .48*** ***p* = .04** N/A

Bold numbers = *p* < .05; N/A = not applicable.

*Linear regression; **Ordinal regression; ***Contingency table for categorical variables (Fisher's exact test).

^a^Analysis of the relation between the nominal (severe and extreme vs. moderate) version of DASS‐anxiety vs. headache‐frequency. Higher headache‐frequency was reported if the level of anxiety was severe to extreme compared to moderate.

^b^Reporting more symptoms (i.e., moderate and hard) of CS was related to higher scores on HIT‐6.

^c^33% of participants with poor sleep quality reported moderate to hard symptoms of CS, compared to 22% with optimal sleep quality reporting subclinical symptoms.

^d^Being highly physical active was related to higher right suboccipital PPTs compared to being moderate physical active.

^e^Shorter (i.e., 7–14 hours/week) screen‐time was related to higher right suboccipital PPTs compared to longer (i.e., >21 hours/week) screen‐time; Δ = right tibialis anterior PPTs were higher with a screen‐time of 7–14 hours compared to >21 hours; ΔΔ = being low physical active was related to lower PPTs on the left tibialis anterior compared to being moderate and high physical active.

^f^Higher CSI‐scores (i.e., moderate and hard) were related to higher DASS‐stress scores (i.e., moderate to extreme). Based on a contingency table, distributions of associations between HIT‐6 and CSI differed significantly (Fisher's exact test *p* < .001) with 38.9% of participants with moderate symptoms of CS reporting headache had a severe impact on quality of life.

^g^Distributions of associations between the CSI and PSQI differed; 44% of participants with optimal sleep quality reported no symptoms of CS.

## 4 DISCUSSION

The aim of this study was to analyze relations between biopsychosocial characteristics: Pain processing, lifestyle, and psychosocial characteristics in participants with CeH. Such biopsychosocial framework is needed to prevent the transition from acute to chronic pain (Borsook et al., 2018). Although scientific literature supports multidimensional patient‐centered interventions when managing headaches, such information is currently lacking for episodic CeH (Peters et al., 2012). The reason might be that CeH is still defined as headache attributed to peripheral disorders of the cervical spine (Headache Classification Committee of the International Headache Society, 2013). This narrow definition does however not cover the multidimensional nature of a pain disorder such as CeH, and might consequently contribute to scarce and inconsistent evidence of therapy success in CeH (Coelho et al., 2019; Côté et al., 2019; Fernandez et al., 2020; Fernández‐De‐Las‐Peñas & Cuadrado, 2014; Gross et al., 2016; Luedtke et al., 2016). Therefore, as a first initiative to fill this gap in knowledge, we conducted an explorative study focusing on the multidimensional character of episodic CeH.

### 4.1 Do participants with episodic CeH show signs and symptoms of disturbed pain processing compared to the control group?

#### 4.1.1 Disturbed pain processing in the CeH group

Mechanical pain hyperalgesia, reflected by decreased PPTs, is commonly used to describe sensory sensitization (Drummond & Knudsen, 2011). We analyzed pain processing by inventorying results on the CSI, and measuring cephalic and extra‐cephalic PPTs. Significantly lower cephalic (suboccipital) PPTs in the CeH group compared to the control group are features of sensitization of the trigeminocervical nucleus (Bezov et al., 2011; Chua et al., 2011). Indications for CS in the CeH group were further supported by the lower extra‐cephalic PPTs compared to the control group, and by 77.8% of participants with CeH reporting at least mild symptoms of CS on the CSI (Neblett et al., 2015). It was already reported that higher scores on the CSI predict higher pain‐related disability in patients with musculoskeletal disorders in a primary care setting (Tanaka et al., 2019). This finding was also reflected by the significant relation (*p* = .006, Fisher's exact test) between more reported symptoms of CS and passive responses (such as rest, analgesic intake or a combination of both) to a headache‐attack by the CeH group in our study. Such passive self‐management was previously reported by patients with chronic CeH in the Akershus study. The latter revealed that patients might have low expectations towards traditional medicine, and that CeH is falsely expected to be self‐manageable (Kristoffersen et al., 2013). The general practitioner or neurologist will only be consulted in case of persistent pain or functional limitations. Such behavior might contribute to the chronification of CeH.

A footnote concerning the CSI is its interpretation. Results on the CSI do not refute or confirm CS, but merely are a representation of general distress (Kregel et al., 2018). This consideration is clinically relevant since exclusively relying on the CSI to confirm CS might increase the risk of false‐positive outcomes, which influences therapy approach.

### 4.2 Do participants with episodic CeH present different lifestyle and/or psychosocial characteristics compared to the control group?

#### 4.2.1 Worse sleep quality, more stress and impact of headache on quality of life in the CeH group

In the CeH group, the reported sleep quality and headache‐related quality of life were worse, and the level of stress higher compared to in the control group. The combination of predominant pain, along with multiple comorbid features such as lowered extra‐cephalic PPTs, stress, sleep problems, and a diminished quality of life in the CeH group might be indicative for an already disturbed pain process in participants with episodic CeH, and supports previous studies reporting that CS is not an exclusive feature of chronic headache (Bernstein & Burstein, 2012; Fumal & Schoenen, 2008; Staffe et al., 2019). Such disturbed pain processing is influenced by top‐down lifestyle and psychosocial risk factors which means that these factors could maintain, and even drive a pain process (Staffe et al., 2019).

Conditioned pain modulation (CPM) and temporal summation should however be assessed in episodic CeH to determine a nociceptive profile (Yarnitsky, 2015). Such profile, which can be either pro‐ or anti‐nociceptive, might provide essential information for future patient‐centered interventions (Lumley et al., 2011).

### 4.3 Are headache, pain processing, lifestyle, and psychosocial characteristics related in both groups?

#### 4.3.1 Lifestyle and psychosocial characteristics relate to pain processing in the CeH group

Thirty multidimensional quality indicators for headache care were previously agreed on (Peters et al., 2012). Although lifestyle and psychosocial factors were identified as such indicators, no study addresses these factors in patients with episodic CeH. In the CeH group, relations were seen between: (1) worse sleep quality, more stress, and more signs and reported symptoms of CS on the one hand and (2) between a lower level of physical activity, lower quality of life, more stress, and signs of peripheral sensitization on the other hand. Presence of both negative psychosocial characteristics such as stress and more symptoms of CS has already been proposed by physiotherapists to contribute to non‐responsiveness to treatment in patients with CeH (Liebert et al., 2013). Further, negative psychosocial variables like depression, anxiety, and distress are among the most robust general predictors for transition from acute to chronic pain, for example tension‐type headache, neck and back pain (Fumal & Schoenen, 2008; Lumley et al., 2011; Mills et al., 2019).

Questioning the patient's self‐reported symptom burden such as headache‐frequency is another quality indicator (Mills et al., 2019). Headache‐frequency is often used to evaluate therapy success within the context of chronification. This outcome measure is additionally used to estimate the degree of sensitization (Adams & Turk, 2015; Buchgreitz et al., 2006; Luedtke et al., 2016). Higher headache‐frequency might lead to an increased pressure pain sensitivity, and with time CS could develop (Buchgreitz et al., 2006). CeH in our study is, based on the range of frequencies (10 to 15.8 days/month), merely an episodic headache. However, potential catalysts for chronification were detected. Both long sitting duration at work and anxiety were independently related to a higher headache‐frequency in the CeH group. Most research on relations between headache‐frequency and CS concerns primary headaches (e.g., tension‐type headache, migraine). However, the novel key findings in our work pointing in the direction of disturbed pain processing, and previous preliminary work suggesting autonomic dysregulation, can be supportive arguments to justify future studies analyzing central nervous system involvement in episodic CeH (Mingels & Granitzer, 2020).

Nevertheless, more research is needed before our results can be transferred to the clinical practice. Based on recent work it is advised to desensitize the central nervous system in patients with CS by designing an individually tailored multimodal treatment plan comprising pain neuroscience education, cognition targeted exercise therapy, sleep management, stress management, and/or dietary intervention (Arendt‐Nielsen et al., 2018; Nijs et al., 2014, 2019; Van Wilgen et al., 2014).

### 4.4 Limitations and suggestions

In this study, several statistical analyses were used requiring some caution when interpreting the results. No Bonferroni correction was applied to analyze simple relations (simple linear regression) among biopsychosocial characteristics themselves, and between these characteristics and headache characteristics. Such correction is not demanded since we examined the effect of one independent variable on the outcome of interest. Thus, only one hypothesis was tested at a time (i.e., no increase in the false positive error rate) (Andrade, 2019). However, caution is needed to interpret and generalize results. Several variables were selected based on priori hypotheses and entered in the regression model, leading to many hypotheses being tested. The backward stepwise approach was used to downsize the model. The VIF was used in case two independent variables were related to the dependent variable.

The rather small sample size (*n* = 36) will tend to overestimate an effect. Post hoc power calculations for most relevant outcomes were: 46.2% PPT suboccipital left, 43% PPT suboccipital right, and 46% CSI. A statistical correct interpretation for results on, for example, the CSI is that the null hypothesis will be erroneously rejected in 54% (=β probability) of the cases, were this study is to be repeated a large number of times. However, we only performed our study once, and no probability can be assigned to a singular, observed result. Thus, we currently have no method for deciding whether this one case was a false‐negative or a true‐negative finding (Levine & Ensom, 2001).

Psychosocial characteristics in participants with episodic CeH should be further explored. More research on maladaptive perceptions is needed. Such perceptions influence a patient's health behavior and contribute to the maintenance of chronic pain (Simpson et al., 2018). Additionally, more attention should be given to positive characteristics that may be protective against chronic pain.

In our study, 33% of participants with episodic CeH and poor sleep quality reported higher degrees of symptoms of CS. Since chronic exposure to insufficient sleep may increase vulnerability to chronic pain by altering processes of nociceptive habituation and sensitization, we recommend to assess additional CPM and temporal summation in patients with episodic CeH (Staffe et al., 2019). Although a decreased CPM and enhanced temporal summation are predictive for pain, to our knowledge, only one study assessed CPM in patients with chronic CeH (Chua et al., 2011). Having a less efficient CPM when being pain‐free at baseline, suggests that upon a pain‐generating event, a patient is at higher risk to develop pain than patients showing an efficient CPM at baseline (Yarnitsky, 2015).

Next, the inconsistency between low extra‐ and cephalic PPTs, and mainly mild to moderate reported symptoms of CS (cfr. CSI) in the CeH group might raise questions. A first consideration concerning this result is that the CSI provides no direct measure of CS, but results rather represent general distress (Neblett et al., 2015). Our results are in line with Kregel et al. (2018). They reported a weaker relation between results on the CSI and measurements of PPT and CPM, compared to a stronger relation between results on the CSI and measurements of pain intensity, quality of life, pain disability, current pain status, and pain catastrophizing (Kregel et al., 2018). Therefore, it seems advised not to use the CSI as a sole screening instrument, but complementary to a comprehensive screening protocol.

## 5 CONCLUSION

Exploring multidimensional factors in CeH demonstrated relations between (1) worse sleep quality, more stress, and more signs and reported symptoms of CS, and between (2) lower level of physical activity, lower quality of life, more stress, and signs of peripheral sensitization. These key findings are novel and fill a gap in current scientific literature on relevant quality indicators for headache care. However, future research is needed to determine a pain profile and to evaluate a patient‐centered intervention based on such profile.

## CONFLICT OF INTEREST

The authors declare that they have no conflict of interest.

### PEER REVIEW

The peer review history for this article is available at https://publons.com/publon/10.1002/brb3.2339

## Supporting information

SUPPORTING INFORMATION

## References

[brb32339-bib-0001] Adams, L. , & Turk, D. (2015). Psychosocial factors and central sensitivity syndromes. Current Rheumatology Reviews, 11(2), 96–108. 26088211 10.2174/1573397111666150619095330 PMC4728142

[brb32339-bib-0002] Alburquerque‐Sendín, F. , Madeleine, P. , Fernández‐De‐Las‐Peñas, C. , Camargo, P. , & Salvini, T. (2018). Spotlight on topographical pressure pain sensitivity maps: A review. Journal of Pain Research, 11, 215–225. 29403305 10.2147/JPR.S135769 PMC5779713

[brb32339-bib-0003] Andrade, C. (2019). Author's response to: Multiple testing and protection against type I error using *p* value correction: Application in cross‐sectional study designs. Indian Journal of Psychological Medicine, 41(2), 198. 30983676 10.4103/IJPSYM.IJPSYM_61_19 PMC6436419

[brb32339-bib-0004] Arendt‐Nielsen, L. , Morlion, B. , Perrot, S. , Dahan, A. , Dickenson, A. , Kress, H. G. , Wells, C. , Bouhassira, D. , & Mohr Drewes, A. (2018). Assessment and manifestation of central sensitisation across different chronic pain conditions. European Journal of Pain, 22(2), 216–241. 29105941 10.1002/ejp.1140

[brb32339-bib-0005] Balaguier, R. , Madeleine, P. , & Vuillerme, N. (2016). Is one trial sufficient to obtain excellent pressure pain threshold reliability in the low back of asymptomatic individuals? A test‐retest study. PLOS One, 11, e0160866. 27513474 10.1371/journal.pone.0160866 PMC4981327

[brb32339-bib-0006] Belgian Headache Society . (2020). belgianheadachesociety.be/bhs/page.asp?lang=en&navid=459&mod=patients&page=calendar

[brb32339-bib-0007] Bernstein, C. , & Burstein, R. (2012). Sensitization of the trigeminovascular pathway: Perspective and implications to migraine pathophysiology. Journal of Clinical Neurology, 8(2), 89–99. 22787491 10.3988/jcn.2012.8.2.89 PMC3391624

[brb32339-bib-0008] Bezov, D. , Ashina, S. , Jensen, R. , & Bendtsen, L. (2011). Pain perception studies in tension‐type headache. Headache, 51(2), 262–271. 21029081 10.1111/j.1526-4610.2010.01768.x

[brb32339-bib-0009] Bialosky, J. E. , Beneciuk, J. M. , Bishop, M. D. , Coronado, R. A. , Penza, C. W. , Simon, C. B. , & George, S. Z. (2018). Unraveling the mechanisms of manual therapy: Modelling an approach. Journal of Orthopaedic and Sports Physical Therapy, 48, 8–18. 10.2519/jospt.2018.7476 29034802

[brb32339-bib-0010] Binderup, A. T. , Arendt‐Nielsen, L. , & Madeleine, P. (2010). Pressure pain sensitivity maps of the neck‐shoulder and the low back regions in men and women. BMC Musculoskeletal Disorders, 11, 234. 20939890 10.1186/1471-2474-11-234 PMC2964538

[brb32339-bib-0011] Borsook, D. , Youssef, A. M. , Simons, L. , Elman, I. , & Eccleston, C. (2018). When pain gets stuck: The evolution of pain chronification and treatment resistance. Pain, 159(12), 2421–2436. 30234696 10.1097/j.pain.0000000000001401 PMC6240430

[brb32339-bib-0012] Buchgreitz, L. , Lyngberg, A. C. , Bendtsen, L. , & Jensen, R. (2006). Frequency of headache is related to sensitization: A population study. Pain, 123(1‐2), 19–27. 16630694 10.1016/j.pain.2006.01.040

[brb32339-bib-0013] Buse, D. C. , Sollars, C. M. , Steiner, T. J. , Jensen, R. H. , Al Jumah, M. A. , & Lipton, R. B. (2012). Why HURT? A review of clinical instruments for headache management. Current Pain and Headache Reports, 16(3), 237–254. 22487847 10.1007/s11916-012-0263-1

[brb32339-bib-0014] Buysse, D. J. , Reynolds, C. F. , Monk, T. H. , Berman, S. R. , & Kupfer, D. J. (1989). The Pittsburgh Sleep Quality Index: A new instrument for psychiatric practice and research. Psychiatry Research, 28(2), 193–213. 2748771 10.1016/0165-1781(89)90047-4

[brb32339-bib-0015] Castien, R. F. , Van Der Wouden, J. C. , & De Hertogh, W. (2018). Pressure pain thresholds over the cranio‐cervical region in headache: A systematic review and meta‐analysis. Journal of Headache and Pain, 19(1), 9. 10.1186/s10194-018-0833-7 PMC5786597 29374331

[brb32339-bib-0016] Chesterton, L. S. , Sim, J. , Wright, C. C. , & Foster, N. E. (2007). Interrater reliability of algometry in measuring pressure pain thresholds in healthy humans, using multiple raters. Clinical Journal of Pain, 23(9), 760–766. 10.1097/AJP.0b013e318154b6ae 18075402

[brb32339-bib-0017] Chua, N. H. L. , Van Suijlekom, H. A. , Vissers, K. C. , Arendt‐Nielsen, L. , & Wilder‐Smith, O. H. (2011). Differences in sensory processing between chronic cervical zygapophysial joint pain patients with and without cervicogenic headache. Cephalalgia, 31(8), 953–963. 21571757 10.1177/0333102411408358

[brb32339-bib-0018] Coelho, M. , Ela, N. , Garvin, A. , Cox, C. , Sloan, W. , Palaima, M. , & Cleland, J. A. (2019). The effectiveness of manipulation and mobilization on pain and disability in individuals with cervicogenic and tension‐type headaches: A systematic review and meta‐analysis. Physical Therapy Reviews, 24(1–2), 29–43.

[brb32339-bib-0019] Côté, P. , Yu, H. , Shearer, H. M. , Randhawa, K. , Wong, J. J. , Mior, S. , Ameis, A. , Carroll, L. J. , Nordin, M. , Varatharajan, S. , Sutton, D. , Southerst, D. , Jacobs, C. , Stupar, M. , Taylor‐Vaisey, A. , Gross, D. P. , Brison, R. J. , Paulden, M. , Ammendolia, C., … Lacerte, M. (2019). Non‐pharmacological management of persistent headaches associated with neck pain: A clinical practice guideline from the Ontario Protocol for Traffic Injury Management (OPTIMa) Collaboration. European Journal of Pain, 23(6), 1051–1070. 30707486 10.1002/ejp.1374

[brb32339-bib-0020] de Beurs, E. , Van Dyck, R. , Marquenie, L. A. , Lange, A. , & Blonk, R. W. B. (2001). De DASS: Een vragenlijst voor het meten van depressie, angst en stress. Gedragstherapie, 34, 35–53.

[brb32339-bib-0021] Drummond, P. D. , & Knudsen, L. (2011). Central pain modulation and scalp tenderness in frequent episodic tension‐type headache. Headache, 51, 375–383. 20946433 10.1111/j.1526-4610.2010.01779.x

[brb32339-bib-0022] Falsiroli Maistrello, L. , Rafanelli, M. , & Turolla, A. (2019). Manual therapy and quality of life in people with headache: Systematic review and meta‐analysis of randomized controlled trials. Current Pain and Headache Reports, 23, 78. 31401702 10.1007/s11916-019-0815-8

[brb32339-bib-0023] Fernandez, M. , Moore, C. , Tan, J. , Lian, D. , Nguyen, J. , Bacon, A. , Christie, B. , Shen, I. , Waldie, T. , Simonet, D. , & Bussières, A. (2020). Spinal manipulation for the management of cervicogenic headache: A systematic review and meta‐analysis. European Journal of Pain, 24(9), 1687–1702. 32621321 10.1002/ejp.1632

[brb32339-bib-0024] Fernández‐De‐Las‐Peñas, C. , & Cuadrado, M. L. (2014). Therapeutic options for cervicogenic headache. Expert Review of Neurotherapeutics, 14(1), 39–49. 24308280 10.1586/14737175.2014.863710

[brb32339-bib-0025] Fernández‐De‐Las‐Peñas, C. , Florencio, L. L. , Plaza‐Manzano, G. , & Arias‐Buría, J. L. (2020). Clinical reasoning behind non‐pharmacological interventions for the management of headaches: A narrative literature review. International Journal of Environmental Research and Public Health, 17(11), 4126. 10.3390/ijerph17114126 PMC7312657 32527071

[brb32339-bib-0026] Finocchietti, S. , Nielsen, M. , Mørch, C. D. , Arendt‐Nielsen, L. , & Graven‐Nielsen, T. (2011). Pressure‐induced muscle pain and tissue biomechanics: A computational and experimental study. European Journal of Pain, 15(1), 36–44. 20591707 10.1016/j.ejpain.2010.05.010

[brb32339-bib-0027] Fumal, A. , & Schoenen, J. (2008). Tension‐type headache: Current research and clinical management. Lancet Neurology, 7(1), 70–83. 18093564 10.1016/S1474-4422(07)70325-3

[brb32339-bib-0028] Global Strategy on Diet, Physical Activity and Health . (2016). Physical activity and adults. World Health Organization. http://who.int/dietphysicalactivity/factsheet_adults/en/

[brb32339-bib-0029] Gross, A. R. , Paquin, J. P. , Dupont, G. , Blanchette, S. , Lalonde, P. , Cristie, T. , Graham, N. , Kay, T. M. , Burnie, S. J. , Gelley, G. , Goldsmith, C. H. , Forget, M. , Santaguida, P. L. , Yee, A. J. , Radisic, G. G. , Hoving, J. L. , & Bronfort, G. (2016). Exercises for mechanical neck disorders: A Cochrane review update. Manual Therapy, 24, 25–45. 27317503 10.1016/j.math.2016.04.005

[brb32339-bib-0030] Headache Classification Committee of the International Headache Society. (2013). The International Classification of Headache Disorders, 3rd edition (beta version). Cephalalgia, 33(9), 629–808. 23771276 10.1177/0333102413485658

[brb32339-bib-0031] Kawata, A. K. , Coeytaux, R. R. , Devellis, R. F. , Finkel, A. G. , Mann, J. D. , & Kahn, K. (2005). Psychometric properties of the HIT‐6 among patients in a headache‐specialty practice. Headache, 45(6), 638–643. 15953295 10.1111/j.1526-4610.2005.05130.x

[brb32339-bib-0032] Koppenhaver, S. L. , Walker, M. J. , Su, J. , Mcgowen, J. M. , Umlauf, L. , Harris, K. D. , & Ross, M. D. (2015). Changes in lumbar multifidus muscle function and nociceptive sensitivity in low back pain patient responders versus non‐responders after dry needling treatment. Manual Therapy, 20(6), 769–776. 25801100 10.1016/j.math.2015.03.003

[brb32339-bib-0033] Kosinski, M. , Bayliss, M. S. , Bjorner, J. B. , Ware, Jr., J. E. , Garber, W. H. , Batenhorst, A. , Cady, R. , Dahlöf, C. G. H. , Dowson, A. , & Tepper, S. (2003). A six‐item short‐form survey for measuring headache impact: The HIT‐6. Quality of Life Research, 12, 963–974. 14651415 10.1023/a:1026119331193

[brb32339-bib-0034] Kregel, J. , Schumacher, C. , Dolphens, M. , Malfliet, A. , Goubert, D. , Lenoir, D. , Cagnie, B. , Meeus, M. , & Coppieters, I. (2018). Convergent validity of the Dutch Central Sensitization Inventory: Associations with psychophysical pain measures, quality of life, disability, and pain cognitions in patients with chronic spinal pain. Pain Practice, 18(6), 777–787. 29222851 10.1111/papr.12672

[brb32339-bib-0035] Kregel, J. , Vuijk, P. J. , Descheemaeker, F. , Keizer, D. , Van Der Noord, R. , Nijs, J. , Cagnie, B. , Meeus, M. , & Van Wilgen, P. (2016). The Dutch Central Sensitization Inventory (CSI): Factor analysis, discriminative power, and test‐retest reliability. Clinical Journal of Pain, 32(7), 624–630. 10.1097/AJP.0000000000000306 26418360

[brb32339-bib-0036] Kristoffersen, E. S. , Lundqvist, C. , Aaseth, K. , Grande, R. B. , & Russell, M. B. (2013). Management of secondary chronic headache in the general population: The Akershus study of chronic headache. Journal of Headache and Pain, 14(1), 5. 10.1186/1129-2377-14-5 PMC3606965 23565808

[brb32339-bib-0037] Levine, M. , & Ensom, M. H. H. (2001). Post hoc power analysis: An idea whose time has passed? Pharmacotherapy, 21(4), 405–409. 11310512 10.1592/phco.21.5.405.34503

[brb32339-bib-0038] Liebert, A. , Rebbeck, T. , Elias, S. , Hawkins, D. , & Adams, R. (2013). Musculoskeletal physiotherapists' perceptions of non‐responsiveness to treatment for cervicogenic headache. Physiotherapy: Theory and Practice, 29(8), 616–629. 23724830 10.3109/09593985.2013.783894

[brb32339-bib-0039] Lluch Girbés, E. , Meeus, M. , Baert, I. , & Nijs, J. (2015). Balancing “hands‐on” with “hands‐off” physical therapy interventions for the treatment of central sensitization pain in osteoarthritis. Manual Therapy, 20, 349–352. 25169787 10.1016/j.math.2014.07.017

[brb32339-bib-0040] Lovibond, P. F. , & Lovibond, S. H. (1995). The structure of negative emotional states: Comparison of the Depression Anxiety Stress Scales (DASS) with the Beck Depression and Anxiety Inventories. Behaviour Research and Therapy, 33(3), 335–343. 7726811 10.1016/0005-7967(94)00075-u

[brb32339-bib-0041] Luedtke, K. , Allers, A. , Schulte, L. H. , & May, A. (2016). Efficacy of interventions used by physiotherapists for patients with headache and migraine‐systematic review and meta‐analysis. Cephalalgia, 36(5), 474–492. 26229071 10.1177/0333102415597889

[brb32339-bib-0042] Lumley, M. A. , Cohen, J. L. , Borszcz, G. S. , Cano, A. , Radcliffe, A. M. , Porter, L. S. , Schubiner, H. , & Keefe, F. J. (2011). Pain and emotion: A biopsychosocial review of recent research. Journal of Clinical Psychology, 67(9), 942–968. 21647882 10.1002/jclp.20816 PMC3152687

[brb32339-bib-0043] Marinus, J. , Visser, M. , Van Hilten, J. J. , Lammers, G. J. , & Stiggelbout, A. M. (2003). Assessment of sleep and sleepiness in Parkinson disease. Sleep, 26(8), 1049–1054. 14746389 10.1093/sleep/26.8.1049

[brb32339-bib-0044] Martin, M. , Blaisdell, B. , Kwong, J. W. , & Bjorner, J. B. (2004). The Short‐Form Headache Impact Test (HIT‐6) was psychometrically equivalent in nine languages. Journal of Clinical Epidemiology, 57(12), 1271–1278. 15617953 10.1016/j.jclinepi.2004.05.004

[brb32339-bib-0045] Martínez‐Segura, R. , De‐La‐Llave‐Rincón, A. I. , Ortega‐Santiago, R. , Cleland, J. A. , & Fernández‐De‐Las‐Peñas, C. (2012). Immediate changes in widespread pressure pain sensitivity, neck pain, and cervical range of motion after cervical or thoracic thrust manipulation in patients with bilateral chronic mechanical neck pain: A randomized clinical trial. Journal of Orthopaedic and Sports Physical Therapy, 42(9), 806–814. 10.2519/jospt.2012.4151 22711239

[brb32339-bib-0046] Matheson, L. N. , Verna, J. , Saunders‐Enright, D. , Gherscovici, E. , Kemp, B. , & Mayer, J. (2020). Development and validation of a method to screen for co‐morbid depression by non‐behavioral health practitioners treating musculoskeletal pain. Work, 67(1), 55–65. 32955474 10.3233/WOR-203252 PMC7683063

[brb32339-bib-0047] Mills, S. E. E. , Nicolson, K. P. , & Smith, B. H. (2019). Chronic pain: A review of its epidemiology and associated factors in population‐based studies. British Journal of Anaesthesia, 123(2), e273–e283. 31079836 10.1016/j.bja.2019.03.023 PMC6676152

[brb32339-bib-0048] Mingels, S. , & Granitzer, M. (2020). Response of the autonomic activity to stress provocation in females with cervicogenic headache compared to asymptomatic controls: A cross‐sectional study. European Journal of Physical and Rehabilitation Medicine, 56(2), 175–183. 31939264 10.23736/S1973-9087.20.05836-0

[brb32339-bib-0049] Mollayeva, T. , Thurairajah, P. , Burton, K. , Mollayeva, S. , Shapiro, C. M. , & Colantonio, A. (2016). The Pittsburgh sleep quality index as a screening tool for sleep dysfunction in clinical and non‐clinical samples: A systematic review and meta‐analysis. Sleep Medicine Reviews, 25, 52–73. 26163057 10.1016/j.smrv.2015.01.009

[brb32339-bib-0050] Moore, C. S. , Sibbritt, D. W. , & Adams, J. (2017). A critical review of manual therapy use for headache disorders: Prevalence, profiles, motivations, communication and self‐reported effectiveness. BMC Neurology, 17, 61. 28340566 10.1186/s12883-017-0835-0 PMC5364599

[brb32339-bib-0051] Neblett, R. , Hartzell, M. M. , Cohen, H. , Mayer, T. G. , Williams, M. , Choi, Y. , & Gatchel, R. J. (2015). Ability of the central sensitization inventory to identify central sensitivity syndromes in an outpatient chronic pain sample. Clinical Journal of Pain, 31(4), 323–332. 10.1097/AJP.0000000000000113 24806467

[brb32339-bib-0052] Neblett, R. , Hartzell, M. M. , Mayer, T. G. , Cohen, H. , & Gatchel, R. J. (2017). Establishing clinically relevant severity levels for the central sensitization inventory. Pain Practice, 17(2), 166–175. 26989894 10.1111/papr.12440

[brb32339-bib-0053] Nijs, J. , Leysen, L. , Vanlauwe, J. , Logghe, T. , Ickmans, K. , Polli, A. , Malfliet, A. , Coppieters, I. , & Huysmans, E. (2019). Treatment of central sensitization in patients with chronic pain: Time for change? Expert Opinion on Pharmacotherapy, 20(16), 1961–1970. 31355689 10.1080/14656566.2019.1647166

[brb32339-bib-0054] Nijs, J. , Malfliet, A. , Ickmans, K. , Baert, I. , & Meeus, M. (2014). Treatment of central sensitization in patients with ‘unexplained’ chronic pain: An update. Expert Opinion on Pharmacotherapy, 15(12), 1671–1683. 24930805 10.1517/14656566.2014.925446

[brb32339-bib-0055] O'sullivan, P. (2012). It's time for change with the management of nonspecific chronic low back pain. British Journal of Sports Medicine, 46, 224–227. 21821612 10.1136/bjsm.2010.081638

[brb32339-bib-0056] O'sullivan, P. , Waller, R. , Wright, A. , Gardner, J. , Johnston, R. , Payne, C. , Shannon, A. , Ware, B. , & Smith, A. (2014). Sensory characteristics of chronic non‐specific low back pain: A subgroup investigation. Manual Therapy, 19(4), 311–318. 24731602 10.1016/j.math.2014.03.006

[brb32339-bib-0057] Parkitny, L. , Mcauley, J. H. , Walton, D. , Pena Costa, L. O. , Refshauge, K. M. , Wand, B. M. , Di Pietro, F. , & Moseley, G. L. (2012). Rasch analysis supports the use of the depression, anxiety, and stress scales to measure mood in groups but not in individuals with chronic low back pain. Journal of Clinical Epidemiology, 65(2), 189–198. 21889306 10.1016/j.jclinepi.2011.05.010

[brb32339-bib-0058] Peters, M. , Jenkinson, C. , Perera, S. , Loder, E. , Jensen, R. , Katsarava, Z. , Gil Gouveia, R. , Broner, S. , & Steiner, T. (2012). Quality in the provision of headache care. 2: Defining quality and its indicators. Journal of Headache and Pain, 13(6), 449–457. 10.1007/s10194-012-0465-2 PMC3464468 22733141

[brb32339-bib-0059] Prushansky, T. , Dvir, Z. , & Defrin‐Assa, R. (2004). Reproducibility indices applied to cervical pressure pain threshold measurements in healthy subjects. Clinical Journal of Pain, 20(5), 341–347. 10.1097/00002508-200409000-00009 15322441

[brb32339-bib-0060] Rosenthal, J. A. (1996). Qualitative descriptors of strength of association and effect size. Journal of Social Service, 21, 37–59.

[brb32339-bib-0061] Scerbo, T. , Colasurdo, J. , Dunn, S. , Unger, J. , Nijs, J. , & Cook, C. (2018). Measurement properties of the central sensitization inventory: A systematic review. Pain Practice, 18, 544–554. 28851012 10.1111/papr.12636

[brb32339-bib-0062] Simpson, N. S. , Scott‐Sutherland, J. , Gautam, S. , Sethna, N. , & Haack, M. (2018). Chronic exposure to insufficient sleep alters processes of pain habituation and sensitization. Pain, 159(1), 33–40. 28891869 10.1097/j.pain.0000000000001053 PMC5832516

[brb32339-bib-0063] Smyth, C. A. (2008). Evaluating sleep quality in older adults: The Pittsburgh Sleep Quality Index can be used to detect sleep disturbances or deficits. American Journal of Nursing, 108(5), 42–50. 10.1097/01.NAJ.0000317300.33599.63 18434798

[brb32339-bib-0064] Staffe, A. T. , Bech, M. W. , Clemmensen, S. L. K. , Nielsen, H. T. , Larsen, D. B. , & Petersen, K. K. (2019). Total sleep deprivation increases pain sensitivity, impairs conditioned pain modulation and facilitates temporal summation of pain in healthy participants. PLOS One, 14(12), e0225849. 31800612 10.1371/journal.pone.0225849 PMC6892491

[brb32339-bib-0065] Tanaka, K. , Murata, S. , Nishigami, T. , Mibu, A. , Manfuku, M. , Shinohara, Y. , Tanabe, A. , & Ono, R. (2019). The central sensitization inventory predict pain‐related disability for musculoskeletal disorders in the primary care setting. European Journal of Pain, 23(9), 1640–1648. 31233655 10.1002/ejp.1443

[brb32339-bib-0066] Tashani, O. A. , Astita, R. , Sharp, D. , & Johnson, M. I. (2017). Body mass index and distribution of body fat can influence sensory detection and pain sensitivity. European Journal of Pain, 21(7), 1186–1196. 28263427 10.1002/ejp.1019

[brb32339-bib-0067] Van Wilgen, P. , Beetsma, A. , Neels, H. , Roussel, N. , & Nijs, J. (2014). Physical therapists should integrate illness perceptions in their assessment in patients with chronic musculoskeletal pain; a qualitative analysis. Manual Therapy, 19(3), 229–234. 24389339 10.1016/j.math.2013.11.006

[brb32339-bib-0068] Varatharajan, S. , Ferguson, B. , Chrobak, K. , Shergill, Y. , Côté, P. , Wong, J. J. , Yu, H. , Shearer, H. M. , Southerst, D. , Sutton, D. , Randhawa, K. , Jacobs, C. , Abdulla, S. , Woitzik, E. , Marchand, A. A. , van der Velde, G. , Carroll, L. J. , Nordin, M. , Ammendolia, C. , … Taylor‐Vaisey, A. (2016). Are non‐invasive interventions effective for the management of headaches associated with neck pain? An update of the Bone and Joint Decade Task Force on Neck Pain and Its Associated Disorders by the Ontario Protocol for Traffic Injury Management (OPTIMa) Collaboration. European Spine Journal, 25(7), 1971–1999. 26851953 10.1007/s00586-016-4376-9

[brb32339-bib-0069] Walton, D. , Macdermid, J. , Nielson, W. , Teasell, R. , Chiasson, M. , & Brown, L. (2011). Reliability, standard error, and minimum detectable change of clinical pressure pain threshold testing in people with and without acute neck pain. Journal of Orthopaedic and Sports Physical Therapy, 41(9), 644–650. 10.2519/jospt.2011.3666 21885906

[brb32339-bib-0070] Woolf, C. J. (2011). Central sensitization: Implications for the diagnosis and treatment of pain. Pain, 152, S2–S15. 20961685 10.1016/j.pain.2010.09.030 PMC3268359

[brb32339-bib-0071] Yarnitsky, D. (2015). Role of endogenous pain modulation in chronic pain mechanisms and treatment. Pain, 156, S24–S31. 25789433 10.1097/01.j.pain.0000460343.46847.58

[brb32339-bib-0072] Ylinen, J. , Nykänen, M. , Kautiainen, H. , & Häkkinen, A. (2007). Evaluation of repeatability of pressure algometry on the neck muscles for clinical use. Manual Therapy, 12(2), 192–197. 16956783 10.1016/j.math.2006.06.010

