# Supplementary material for: Exploring multidimensional characteristics in cervicogenic headache: Relations between pain processing, lifestyle, and psychosocial factors
Source: Brain Behav. 2021 Sep 2;11(10):e2339. doi: 10.1002/brb3.2339 (PMC8553329; doi:10.1002/brb3.2339)
Supplement: Supplementary file 1 — SUPPORTING INFORMATION [file BRB3-11-e2339-s001.docx]

**Supplementary material**

**Appendix a –** **Relations between independent variables and outcomes**

Relations between age, BMI, their interaction (age*BMI), level of education and employment, and outcomes (headache, pain processing, lifestyle, and psychosocial characteristics) were analysed through multiple linear regression (continuous outcome) or ordinal (logistic) regression (categorical outcome). An overview of the statistical significance (p-values) of these effects in the CeH-group and control-group is provided in Tables a.1 and a.2, respectively.

**Table a.1.** Summary of relations between independent variables and outcomes in the CeH-group (n = 18)

| Outcome | Age (p) | BMI (p) | Age x BMI (p) | LOE (p) | Job (p) |
| --- | --- | --- | --- | --- | --- |
| Headache characteristics | | | | | |
| 100 mm VAS | .66 | .13 | .63 | .63 | .63 |
| Pain processing characteristics | | | | | |
| CSI | .36 | .49 | .16 | .05 | .05 |
| PPT sub L | .17 | .82 | .41 | .47 | .47 |
| PPT sub R | .08 | .48 | .27 | .27 | .27 |
| PPT ES L | .67 | .38 | .81 | .4 | .4 |
| PPT ES R | .86 | .75 | .63 | .54 | .54 |
| PPT Tib L | .33 | .94 | .78 | .89 | .89 |
| PPT Tib R | .86 | .75 | .63 | .59 | .59 |
| Lifestyle characteristics | | | | | |
| PSQI | .3 | .46 | .74 | .3 | .17 |
| Physical activity | .05 | .13 | .41 | .07 | .12 |
| Screen-time | .99 | .5 | .79 | .5 | .06 |
| Sedentary-time work | .07 | .15 | .1 | .59 | .59 |
| Sedentary-time free | .34 | .15 | .67 | .5 | .89 |
| Psychosocial characteristics | | | | | |
| DASS-depression | .62 | .07 | .25 | .77 | .26 |
| DASS-anxiety | .78 | .61 | .95 | .72 | **.02^1^** |
| DASS-stress | .25 | .1 | .51 | .16 | .82 |
| HIT-6 | .6 | .76 | .36 | **.007^2^** | **.01^2^** |

LOE = Level of Education; Job = Employment; ES = Erector Spine L1; Tib = Tibialis anterior; L = Left; R = Right; Bold numbers = p < .05; ^1^ = employment (independent, n = 2) was related to higher scores on the DASS-anxiety; ^2^ = employment and level of education (student, n = 2 and high school n = 2) were related to higher scores on the HIT-6.

**Table a.2.** Summary of relations between independent variables and outcomes in the control-group (n = 18)

| Outcome | Age (p) | BMI (p) | Age x BMI (p) | LOE (p) | Job (p) |
| --- | --- | --- | --- | --- | --- |
| Pain processing characteristics | | | | | |
| CSI | .08 | .48 | .27 | .38 | .27 |
| PPT sub L | .18 | .75 | .76 | .48 | .25 |
| PPT sub R | .6 | .38 | .3 | .59 | .35 |
| PPT ES L | .11 | .41 | .45 | .08 | .49 |
| PPT ES R | .32 | .58 | .34 | .09 | .53 |
| PPT Tib L | .66 | .1 | .87 | .16 | .75 |
| PPT Tib R | .58 | .24 | .22 | .14 | .5 |
| Lifestyle characteristics |  |  |  |  |  |
| PSQI | .17 | .82 | .41 | .49 | .91 |
| Physical activity | .41 | .52 | .39 | .45 | .62 |
| Screen-time | .75 | .38 | .23 | .12 | .37 |
| Sedentary-time work | .09 | .05 | .57 | .07 | .57 |
| Sedentary-time free | .61 | .4 | .14 | .12 | .42 |
| Psychosocial characteristics | | | | | |
| DASS-depression | .08 | .45 | .12 | .73 | .74 |
| DASS-anxiety | .22 | .72 | .35 | .83 | .9 |
| DASS-stress | .71 | .6 | .96 | .79 | .77 |

LOE = Level of Education; Job = Employment; ES = Erector Spine L1; Tib = Tibialis anterior; L = Left; R = Right.
